# Supplementary figures and images for: Screening for variable drug responses using human iPSC cohorts
Source: PLoS One. 2025 May 30;20(5):e0323953. doi: 10.1371/journal.pone.0323953 (PMC12124524; doi:10.1371/journal.pone.0323953)

**A**

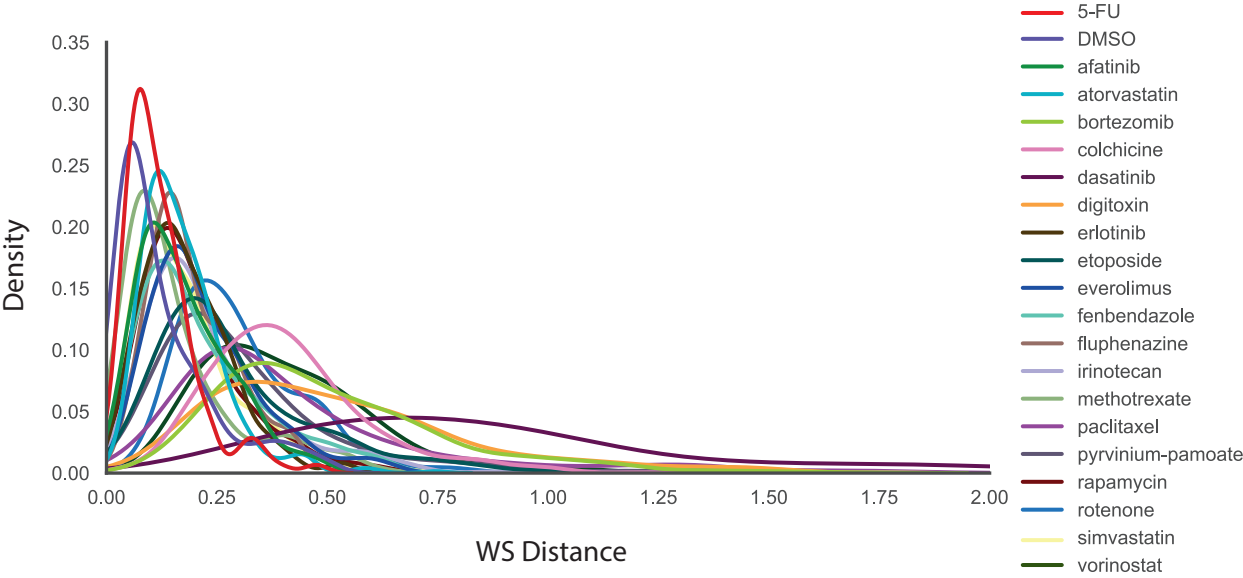

**B**

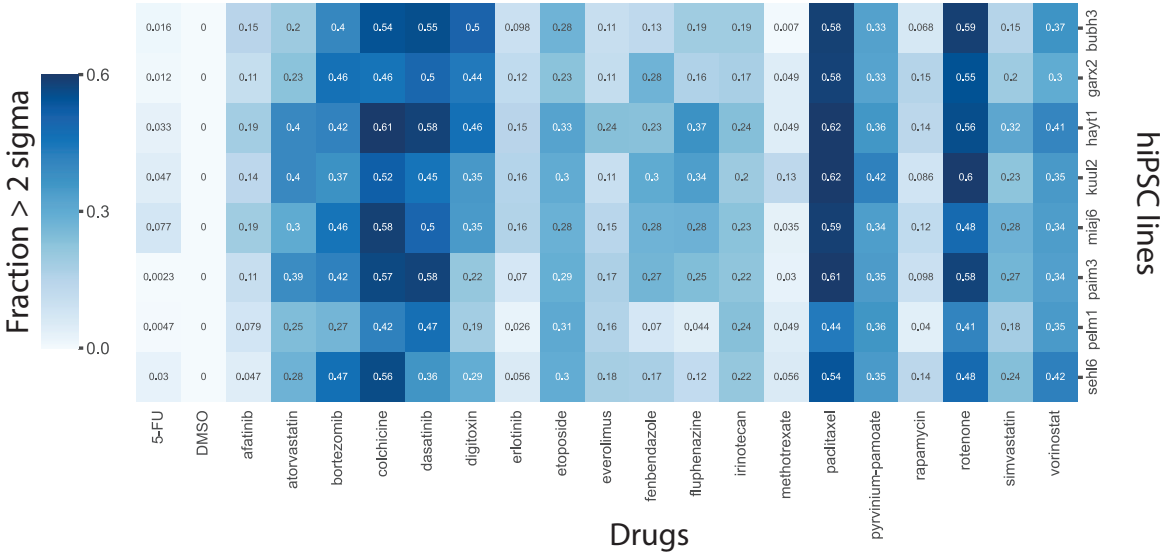

**C**

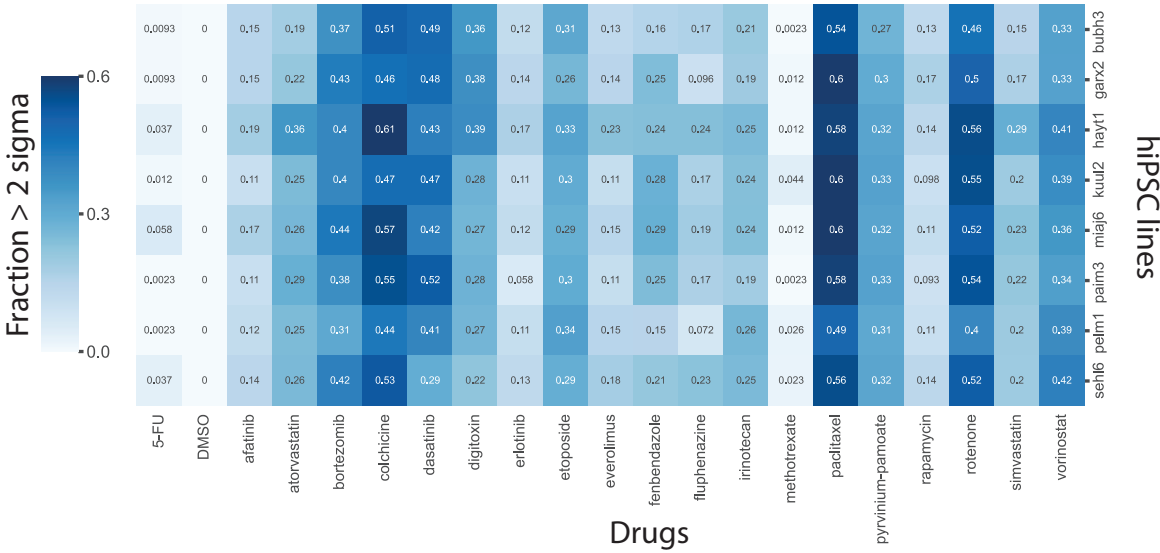

Supplement: S2 Fig — The data for these assays were collected in February and October 2021 respectively, on two different imaging systems (InCell 2200 and CV700 high content imagers). The Wasserstein distance provides a metric of similarity of two distribution [28]. Wasserstein distances of features for each drug-donor pair were calculated and the frequency distribution of the medians of the features for each drug was then plotted. Different drugs show different distributions, with cytotoxic drugs (e.g., bortezimib, dasatinib, paclitaxel and colchicine) showing higher Wasserstein distances, i.e., great variation between features in the two different runs. (B) and (C) Induction plots of drugs and donors that were common across the two assays show in S1B-D Fig. Values in the heatmap are calculated induction values using a cutoff of 2 sigma (same is in Fig 2A). (PDF) [file pone.0323953.s002.pdf]

A

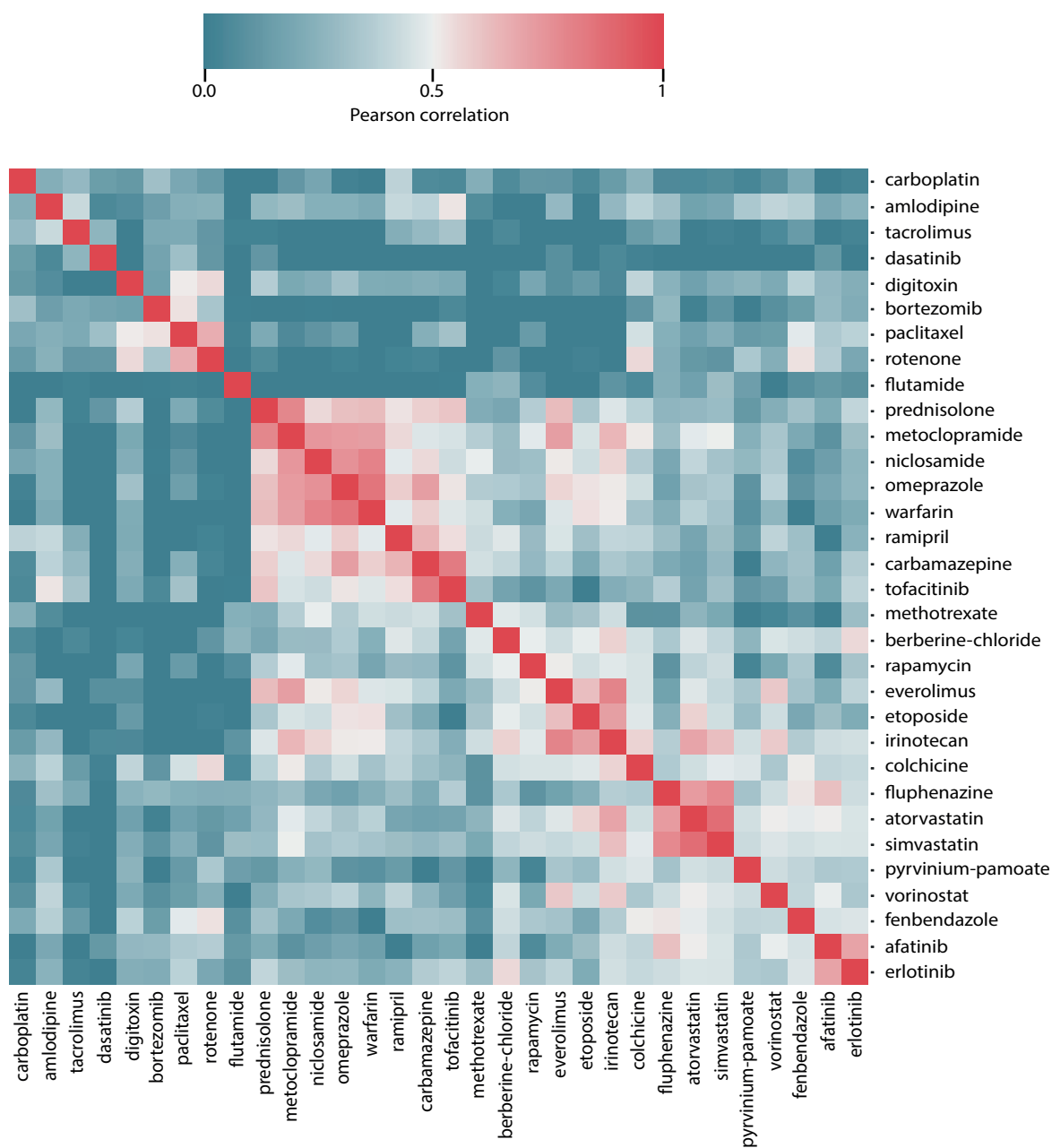

S5 Figure

Supplement: S5 Fig — Note the clusters formed based on the patterns of induction across the donor cohort, suggesting similar responses between different drugs. (PDF) [file pone.0323953.s005.pdf]
