# Supplementary material for: Screening for variable drug responses using human iPSC cohorts
Source: PLoS One. 2025 May 30;20(5):e0323953. doi: 10.1371/journal.pone.0323953 (PMC12124524; doi:10.1371/journal.pone.0323953)
Supplement: S3 Fig — Cell lines used for proteomic analysis are shown in grey boxes. (B) Table of nearest neighbour analysis. Spearman correlation filtered features for all drugs were used to calculate Euclidean distance from DMSO. Induction mean, standard deviation and coefficient of variation (CoV) for each drug are shown. (PDF) [file pone.0323953.s003.pdf]

A

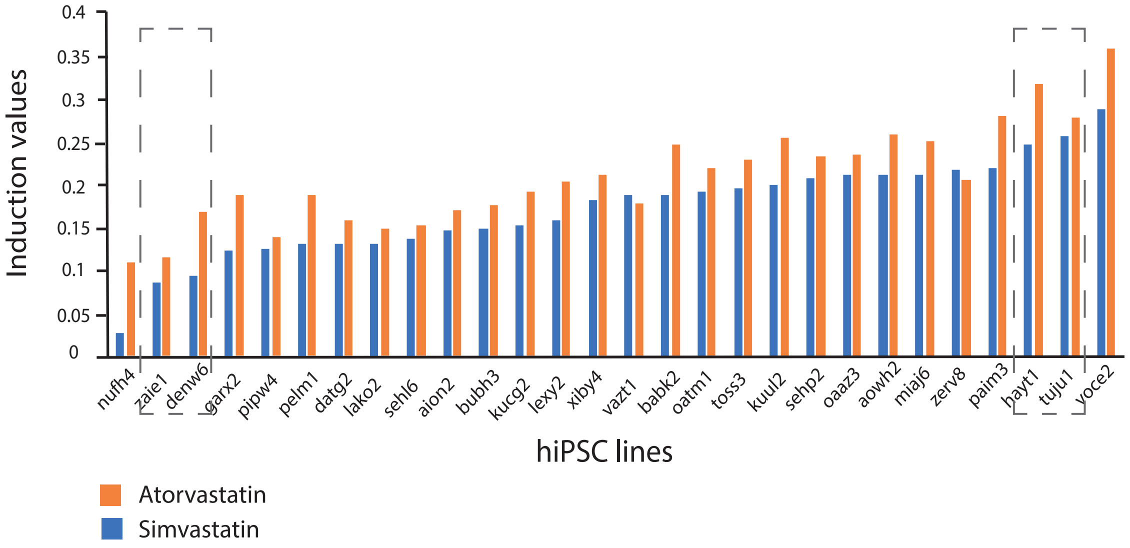
Induction values
0.4
0.35
0.3
0.25
0.2
0.15
0.1
0.05
0
nufh4 zaie1 denw6 garx2 pipw4 pelm1 datg2 lako2 sehl6 aion2 bubh3 kucg2 lexy2 xiby4 vazt1 babk2 oatm1 toss3 kuul2 sehp2 oaaz3 aowh2 miaj6 zerv8 paim3 hayt1 tuju1 voce2
hiPSC lines
Atorvastatin
Simvastatin

B

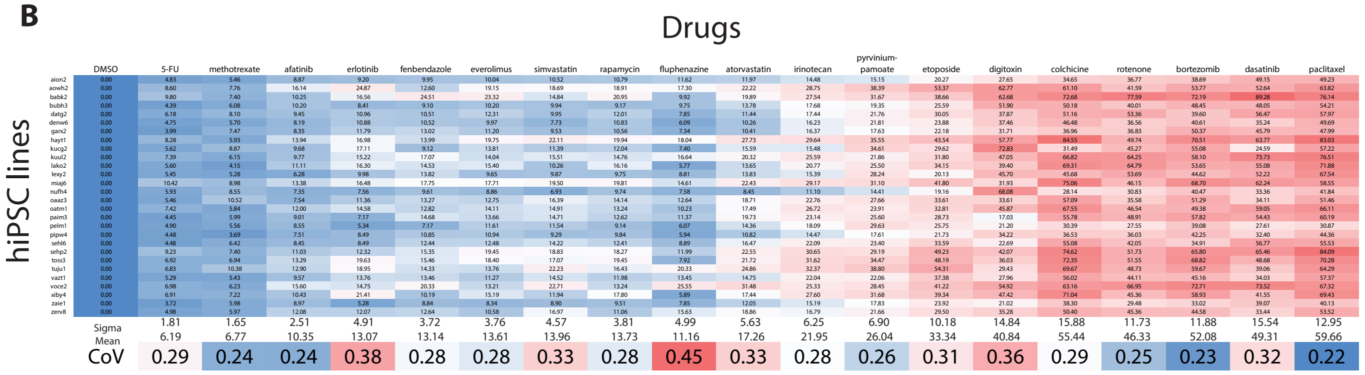
Drugs
hiPSC lines
DMSO 5-FU methotrexate afatinib erlotinib fenbendazole everolimus simvastatin rapamycin fluphenazine atorvastatin irinotecan pyrvinium-pamoate etoposide digitoxin colchicine rotenone bortezomib dasatinib paclitaxel
aion2 aowh2 babk2 bubh3 datg2 denw6 garx2 hayt1 kucg2 kuul2 lako2 lexy2 miaj6 nufh4 oaaz3 oatm1 paim3 pelm1 pipw4 sehl6 sehp2 toss3 tuju1 vazt1 voce2 xiby4 zaie1 zerv8
Sigma 1.81 1.65 2.51 4.91 3.72 3.76 4.57 3.81 4.99 5.63 6.25 6.90 10.18 14.84 15.88 11.73 11.88 15.54 12.95
Mean 6.19 6.77 10.35 13.07 13.14 13.61 13.96 13.73 11.16 17.26 21.95 26.04 33.34 40.84 55.44 46.33 52.08 49.31 59.66
CoV 0.29 0.24 0.24 0.38 0.28 0.28 0.33 0.28 0.45 0.33 0.28 0.26 0.31 0.36 0.29 0.25 0.23 0.32 0.22
