# Supplementary material for: Screening for variable drug responses using human iPSC cohorts
Source: PLoS One. 2025 May 30;20(5):e0323953. doi: 10.1371/journal.pone.0323953 (PMC12124524; doi:10.1371/journal.pone.0323953)
Supplement: S4 Fig — High response lines: tuju1 and hayt1. Low response lines: denw6 and zaie1. Protein FC and p value for each cell line were calculated as in Fig 3. Sample runs from drug treatment set (n = 6) were considered as one group and the DMSO control set (n = 6) was considered as a separate group. Members of cholesterol biosynthesis pathway are represented as purple dots and listed on the right side of the graph. (B) Heat map analysis of proteins showing differential expression (log2FChigh-low > 0.263) between high and low response hiPSC lines following simvastatin treatment. Heatmap generation steps: The protein fold change (FChigh) and p value (Phigh) for high response cell lines were calculated with sample runs from both high response cell lines to Atorvastatin set (n = 6) considered as one group and the DMSO control set (n = 6) considered as a separate group. The protein fold change (FClow) and p value (Plow) for low response cell lines were also calculated. Only proteins with both Phigh ≤0.05 and Plow ≤ 0.05 were considered further, to compare the difference between high and low response. log2FC of these filtered proteins was calculated as (log2FChigh-low = log2FChigh- log2FClow). Heatmap shows FC between drug and DMSO of filtered list z-score normalised. StringDB analysis of filtered proteins shown in (B) in high response lines (C) and low response lines (D). (PDF) [file pone.0323953.s004.pdf]

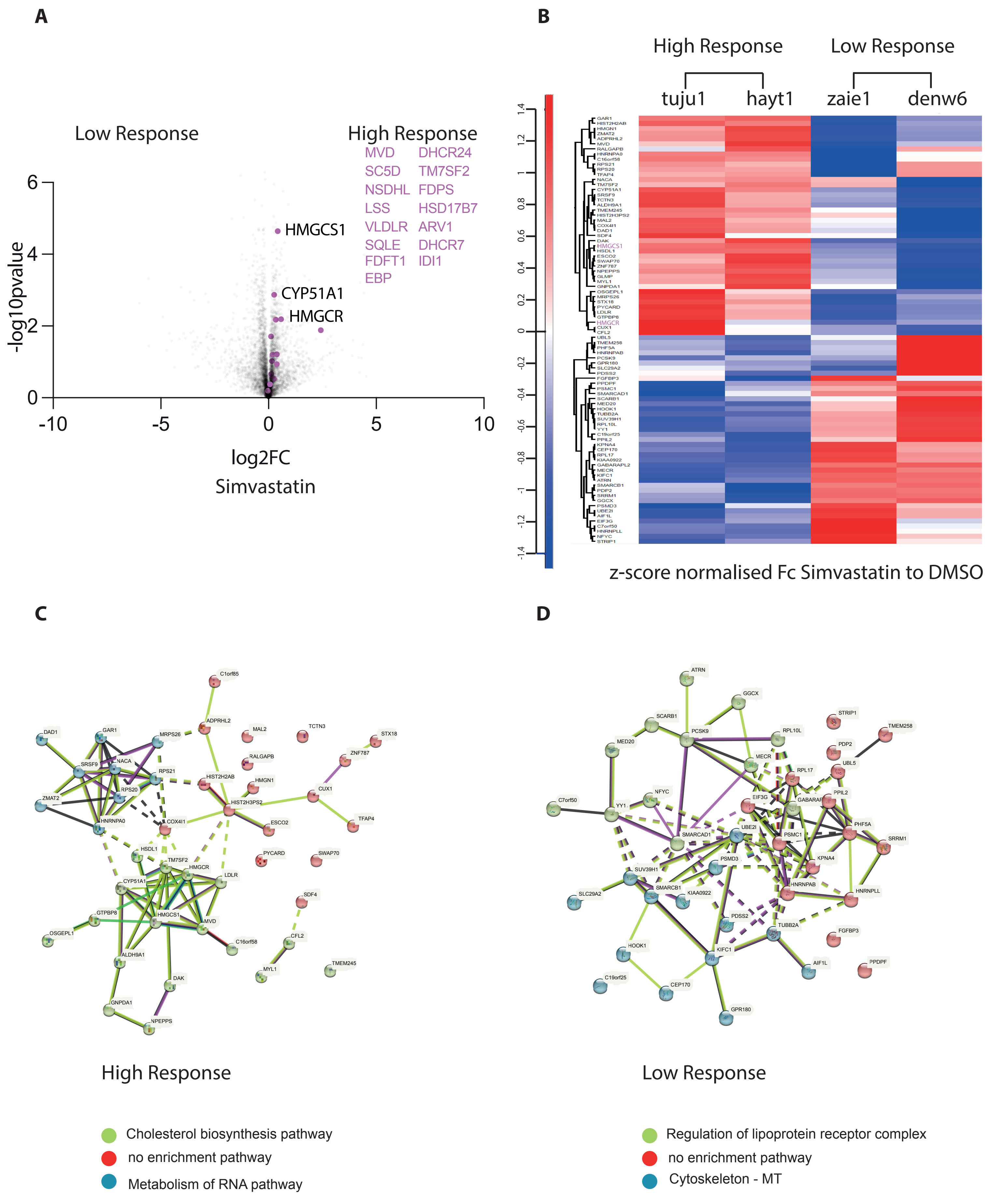
A
Low Response
High Response
MVD
DHCR24
SC5D
TM7SF2
NSDHL
FDPS
LSS
HSD17B7
VLDLR
ARV1
SQLE
DHCR7
FDFT1
IDI1
EBP
HMGCS1
CYP51A1
HMGCR
-log10pvalue
log2FC
Simvastatin
B
High Response
Low Response
tuju1
hayt1
zaie1
denw6
z-score normalised Fc Simvastatin to DMSO
C
High Response
Cholesterol biosynthesis pathway
no enrichment pathway
Metabolism of RNA pathway
D
Low Response
Regulation of lipoprotein receptor complex
no enrichment pathway
Cytoskeleton - MT
