# Supplementary material for: Screening for variable drug responses using human iPSC cohorts
Source: PLoS One. 2025 May 30;20(5):e0323953. doi: 10.1371/journal.pone.0323953 (PMC12124524; doi:10.1371/journal.pone.0323953)
Supplement: S1 Table — (PDF) [file pone.0323953.s006.pdf]

Supplemental Table 1: List of hiPSC lines used in this study

Cell ID	Donor Description	Run #2021-02	Run #2021-10	Run #2023-12
aion2	White British, Male	✓		
aowh2	White British, Female	✓		
babk2	White British, Female	✓		
bubh3	White British, Female	✓		
burb1	White British, Male		✓	✓
datg2	White British, Female	✓		
denw6	White British, Male	✓		✓
deyz2	White British, Female			✓
fiaj1	White British, Male			✓
garx2	White British, Female	✓	✓	
hayt1	White Other, Male	✓	✓	✓
hiaf1	White British, Male			✓
kucg2	White British, Male	✓		
kuul2	White British, Male	✓	✓	
lako2	White British, Female	✓		
lexy2	White British, Female	✓		
melw1	White British, Male			✓
miaj6	White British, Male	✓	✓	
nufh4	White British, Female	✓		
oaaz3	White British, Male	✓		
oatm1	White British, Male	✓		
paim3	White British, Male	✓	✓	
pelm1	White British, Female	✓	✓	
pipw4	White British, Male	✓		
podx2	White British, Female			✓
puhk2	White British, Female			✓
romx1	White British, Male			✓
sebn3	White British, Female			✓
sehl6	White British, Female	✓	✓	
sehp2	White Other, Female	✓		
sohd3	White British, Female			✓
tert1	White British, Male			✓
toss3	White British, Male	✓		
tuju1	White British, Female	✓		✓
vaka5	White British, Female			✓
vazt1	White British, Male	✓		
voce2	White British, Male	✓		
wibj2	White British, Female			✓
xiby4	White British, Female	✓		
zaie1	White British, Female	✓		
zapk3	White British, Male			✓
zerv8	White British, Female	✓		
zoxy3	White British, Female			✓
