# Supplementary material for: Screening for variable drug responses using human iPSC cohorts
Source: PLoS One. 2025 May 30;20(5):e0323953. doi: 10.1371/journal.pone.0323953 (PMC12124524; doi:10.1371/journal.pone.0323953)
Supplement: S2 Table — List of drugs, disease targets and concentrations used in this study. (PDF) [file pone.0323953.s007.pdf]

Supplemental Table 2: List of drugs and concentrations used in this study

Drug	Disease target	Concentration
**DMSO (control)**	*Control*	5 µM
**5-FU**	Cancer drug	5 µM
**Abacavir**	HIV reverse transcriptase inhibitor	5 µM
**Afatinib**	Cancer drug (breast)	5 µM
**Amlodipine**	Heart disease	5 µM
**Atorvastatin**	Heart disease (coronary)	5 µM
**Azathioprine**	Immunosuppressant	5 µM
**Berberine Chloride**	Transcription/mitochondrial function	2.68 µM
**Bortezomib**	Cancer drug	5 µM
**Bosentan**	Heart disease (Pulmonary hypertension)	5 µM
**Carbamazepine**	Epilepsy	5 µM
**Carboplatin**	Cancer drug (neoplasm/carcinoma)	5 µM
**Cyclophosphamide Monohydrate**	Cancer drug (breast neoplasm)	5 µM
**Dasatinib**	Cancer drug	5 µM
**Dexamethasone**	Lung condition	5 µM
**Digitoxin**	Cancer drug	5 µM
**Erlotinib**	Cancer drug (breast)	5 µM
**Ethacrynic Acid**	Cancer drug, ALS, Parkinson’s	5 µM
**Etoposide**	Cancer drug (topoisomerase inhibitor)	1.7 µM
**Everolimus**	Cancer drug (breast)	5 µM
**Fenbendazole**	Parasitic infections	3.34 µM
**Fluphenazine**	Antipsychotic	5 µM
**Flutamide**	Cancer drug (prostate)	5 µM
**Gefitinib**	Cancer drug (breast)	5 µM
**Hydrocortisone**	Steroid	5 µM
**Imatinib**	Cancer drug	5 µM
**Irinotecan**	Cancer drug (topoisomerase inhibitor)	5 µM
**Lansoprazole**	Stomach ulcer	5 µM
**Metformin**	Diabetes	5 µM
**Methotrexate**	Cancer drug (lymphoma)	5 µM
**Metoclopramide**	Nausea/vomiting	5 µM
**Milrinone**	Heart disease (heart failure)	5 µM
**Niclosamide**	Cancer/bacterial/viral infections	5 µM
**Omeprazole**	Stomach ulcer	5 µM
**Paclitaxel**	Cancer drug (breast)	5 µM
**Paroxetine**	Depressive disorder	5 µM
**Prednisolone**	Carotid stenosis	5 µM
**Procaine**	Anaesthesia	5 µM

**Pyrvinium Pamoate**	Antihelminthic drug - anticancer	5 µM
**Ramipril**	Heart disease	5 µM
**Rapamycin**	mTOR inhibitor	5 µM
**Rosiglitazone**	Diabetes	5 µM
**Rotenone**	Pesticide	5 µM
**Salbutamol**	Bronchodilator	5 µM
**Simvastatin**	Coronary disease	5 µM
**Sorafenib**	Cancer drug (breast)	5 µM
**Tacrolimus**	Immunosuppressant	5 µM
**Tofacitinib**	Arthritis	5 µM
**Voriconazole**	Anti-fungal	5 µM
**Vorinostat**	HDAC inhibitor	5 µM
**Warfarin**	Heart disease	5 µM
