# Supplementary material for: Screening for variable drug responses using human iPSC cohorts
Source: PLoS One. 2025 May 30;20(5):e0323953. doi: 10.1371/journal.pone.0323953 (PMC12124524; doi:10.1371/journal.pone.0323953)
Supplement: S3 Table — GO enrichment analysis of top 5% proteins showing increased expression in all four hiPSC lines used for proteomic analysis following atorvastatin treatment. Note pathways related to lipid metabolism. (PDF) [file pone.0323953.s008.pdf]

Supplemental Table 3: GO enrichment analysis of top 5% proteins showing increased expression in all four hiPSC lines used for proteomic analysis following atorvastatin treatment.

GO term	Description	p-value	FDR q-value	Enrichment
GO:0016126	sterol biosynthetic process	1.71E-20	1.37E-16	16.74
GO:0008610	lipid biosynthetic process	5.99E-20	2.39E-16	8.06
GO:0006695	cholesterol biosynthetic process	3.56E-19	9.47E-16	16.59
GO:1902653	secondary alcohol biosynthetic process	3.56E-19	7.11E-16	16.59
GO:0016125	sterol metabolic process	9.72E-19	1.55E-15	12.47
GO:0006694	steroid biosynthetic process	6.08E-18	8.10E-15	12.66
GO:0008202	steroid metabolic process	1.00E-17	1.14E-14	9.85
GO1902652	secondary alcohol metabolic process	1.54E-17	1.54E-14	12.23
GO:000820	cholesterol metabolic process	1.54E-17	1.37E-14	12.23
GO:0006629	lipid metabolic process	1.94E-16	1.55E-13	4.96
Go:0046165	alcohol biosynthetic process	7.83E-15	5.69E-12	11.25
GO:1901617	organic hydroxy compound biosynthetic process	2.03E-14	1.35E-11	9.85
GO:0019216	regulation of lipid metabolic process	2.55E-14	1.57E-11	7.72
GO:0046890	regulation of lipid biosynthetic process	6.87E-14	3.92E-11	10.17
GO:0050810	regulation of steroid biosynthetic process	8.11E-13	4.32E-10	12.19
GO:0006066	alcohol metabolic process	1.02E-12	5.07E-10	7.54
GO:1901615	organic hydroxy compound metabolic process	1.40E-12	6.59E-10	6.46
GO:1902930	regulation of alcohol biosynthetic process	1.85E-12	8.19E-10	13.13
GO:0045540	regulation of cholesterol biosynthetic process	1.85E-12	7.76E-10	13.13
GO:0090181	regulation of cholesterol metabolic process	1.85E-12	7.37E-10	13.13
GO:0106118	regulation of sterol biosynthetic process	1.85E-12	7.02E-10	13.13
