# Supplementary material for: Screening for variable drug responses using human iPSC cohorts
Source: PLoS One. 2025 May 30;20(5):e0323953. doi: 10.1371/journal.pone.0323953 (PMC12124524; doi:10.1371/journal.pone.0323953)
Supplement: S4 Table — (PDF) [file pone.0323953.s009.pdf]

Supplemental Table 4: GO enrichment analysis of top 5% proteins showing increased expression in all four hiPSC lines used for proteomic analysis following simvastatin treatment.

GO Term	Description	P- value	FDR q-value	Enrichment
GO:0016125	sterol metabolic process	6.66E-16	7.50E-13	10.483
GO:1902652	secondary alcohol metabolic process	6.66E-16	7.50E-13	10.483
GO:0008203	cholesterol metabolic process	1.02E-14	5.67E-12	10.207
GO:0008202	steroid metabolic process	1.49E-14	5.67E-12	8.325
GO:0016126	sterol biosynthetic process	1.51E-14	5.67E-12	11.1
GO:1902653	secondary alcohol biosynthetic process	1.51E-14	5.67E-12	11.1
GO:0090181	regulation of cholesterol metabolic process	3.34E-14	1.08E-11	13.361
GO:0019218	regulation of steroid metabolic process	1.06E-13	2.98E-11	11.261
GO:0008610	Lipid biosynthetic process	1.30E-13	3.26E-11	5.9769
GO:0006694	steroid biosynthetic process	1.54E-13	3.46E-11	8.9697
GO:0006695	cholesterol biosynthetic process	2.45E-13	5.02E-11	10.792
GO:0006066	alcohol metabolic process	3.35E-13	6.29E-11	7.8625
GO:1901615	organic hydroxy compound metabolic process	4.83E-13	8.37E-11	7.0851
GO:0045540	regulation of cholesterol biosynthetic process	6.13E-13	8.63E-11	13.059
GO:0106118	regulation of sterol biosynthetic process	6.13E-13	8.63E-11	13.059
GO:1902930	regulation of alcohol biosynthetic process	6.13E-13	8.63E-11	13.059
GO:0050810	regulation of steroid biosynthetic process	7.13E-13	9.45E-11	11.452
GO:1901617	organic hydroxy compound biosynthetic process	8.70E-13	1.09E-10	8.2222
GO:0046165	alcohol biosynthetic process	1.13E-12	1.34E-10	8.9516
GO:0046890	regulation of lipid biosynthetic process	8.12E-12	9.15E-10	8.931
GO:0044283	small molecule biosynthetic process	3.45E-11	3.70E-09	5.3258
GO:0006629	lipid metabolic process	4.54E-11	4.65E-09	4.0913
GO:0062012	regulation of small molecule metabolic process	4.81E-10	4.71E-08	7
GO:0019216	regulation of lipid metabolic process	1.64E-09	1.54E-07	6.475
