# Supplementary material for: Screening for variable drug responses using human iPSC cohorts
Source: PLoS One. 2025 May 30;20(5):e0323953. doi: 10.1371/journal.pone.0323953 (PMC12124524; doi:10.1371/journal.pone.0323953)
Supplement: S5 Table — GO Enrichment Analysis of gene sets/pathways and their associated p-values for highly expressed proteins (Fig 4 heat map (B) values >0.2) of high response lines following atorvastatin treatment. (PDF) [file pone.0323953.s010.pdf]

Supplemental Table 5: GO Enrichment Analysis of gene sets/pathways and their associated p-values for highly expressed proteins (Fig 4 heat map (B) values >0.2) of <u>High response</u> lines following atorvastatin treatment.

Gene set	Description	Ratio	p-value	FDR
GO:0090181	Regulation of cholesterol metabolic process	19.427	4.6062e-7	0.0018237
GO:0008203	cholesterol metabolic process	12.592	0.0000010172	0.0018237
GO:0046890	Regulation of lipid biosynthetic process	12.395	0.0000011345	0.0018237
GO:1902652	Secondary alcohol metabolic process	12.204	0.0000012630	0.0018237
GO:0006695	cholesterol biosynthetic process	15.454	0.0000018865	0.0018237
GO:0016125	Sterol metabolic process	11.497	0.0000019057	0.0018237
GO:1902653	Secondary alcohol biosynthetic process	15.110	0.0000021623	0.0018237
GO:0016126	Sterol biosynthetic process	14.467	0.0000028127	0.0018452
GO:0019218	Regulation of steroid metabolic process	14.467	0.0000028127	0.0018452
GO:0045540	Regulation of cholesterol biosynthetic process	18.278	0.0000062092	0.0030549
