# Supplementary material for: Screening for variable drug responses using human iPSC cohorts
Source: PLoS One. 2025 May 30;20(5):e0323953. doi: 10.1371/journal.pone.0323953 (PMC12124524; doi:10.1371/journal.pone.0323953)
Supplement: S6 Table — (PDF) [file pone.0323953.s011.pdf]

Supplemental Table 6: GO Enrichment Analysis of gene sets/pathways and their associated p-values for highly expressed proteins (Fig 4 heat map (B) values >0.2) of low response lines following atorvastatin treatment.

Gene set	Description	Ratio	p-value	FDR
GO:0043687	Post-translational protein modification	6.8556	3.6169e-7	0.0021354
GO:0006814	Sodium ion transport	9.7013	0.00014502	0.39546
GO:0043062	Extracellular structure organization	5.6680	0.00020094	0.39546
GO:0035752	Sodium ion transmembrane trasnport	11.752	0.00033453	0.49377
GO:0098869	Cellular oxidant detoxification	9.3485	0.00081295	0.51703
GO:0034368	Protein-lipid complex remodeling	41.133	0.00091104	0.51703
GO:0034369	Plasma lipoprotein particle remodeling	41.133	0.00091104	0.51703
GO:0034375	High-density lipoprotein particle remodeling	41.133	0.00091104	0.51703
GO:2000644	Regulation of receptor catabolic process	41.133	0.00091104	0.51703
GO:1905906	Regulation of amyloid fibril formation	41.133	0.00091104	0.51703
