# Supplementary material for: Screening for variable drug responses using human iPSC cohorts
Source: PLoS One. 2025 May 30;20(5):e0323953. doi: 10.1371/journal.pone.0323953 (PMC12124524; doi:10.1371/journal.pone.0323953)
Supplement: S7 Table — GO Enrichment Analysis of gene sets/pathways and their associated p-values for highly expressed proteins (S4 Fig heat map (B) values >0.2) of high response lines following simvastatin treatment. (PDF) [file pone.0323953.s012.pdf]

Supplemental Table 7: GO Enrichment Analysis of gene sets/pathways and their associated p-values for highly expressed proteins (Supp Fig 2 heat map (B) values >0.2) of High response lines following simvastatin treatment.

Gene set	Description	Ratio	p-value	FDR
GO:0090181	Regulation of cholesterol metabolic process	25.728	8.0828e-8	0.00047721
GO:0019218	Regulation of steroid metabolic process	19.159	5.0470e-7	0.0014899
GO:0045540	Regulation of cholesterol biosynthetic process	24.207	0.0000014877	0.0017567
GO:0106118	Regulation of sterol biosynthetic process	24.207	0.0000014877	0.0017567
GO:1902930	Regulation of alcohol biosynthetic process	24.207	0.0000014877	0.0017567
GO:0008203	Cholesterol metabolic process	14.293	0.0000029569	0.0026274
GO:0046890	Regulation of lipid biosynthetic process	14.070	0.0000032471	0.0026274
GO:1902652	Secondary alcohol metabolic process	13.854	0.0000035601	0.0026274
GO:0050810	Regulation of steroid biosynthetic process	19.241	0.0000048505	0.0029923
GO:0016125	Sterol metabolic process	13.051	0.0000050683	0.0029923
