# Supplementary material for: Screening for variable drug responses using human iPSC cohorts
Source: PLoS One. 2025 May 30;20(5):e0323953. doi: 10.1371/journal.pone.0323953 (PMC12124524; doi:10.1371/journal.pone.0323953)
Supplement: S8 Table — GO Enrichment Analysis of gene sets/pathways and their associated p-values for highly expressed proteins (S4 Fig heat map (B) values >0.2) of low response lines following simvastatin treatment. (PDF) [file pone.0323953.s013.pdf]

Supplemental Table 8: GO Enrichment Analysis of gene sets/pathways and their associated p-values for highly expressed proteins (Supp Fig 2 heat map (B) values >0.2) of low response lines following simvastatin treatment.

Gene set	Description	Ratio	p-value	FDR
GO:0036123	Histone H3-K9 dimethylation	58.453	0.00045016	1
GO:0018027	Peptidyl-lysine dimethylation	29.226	0.0019824	1
GO:0036124	Histone H3-K9 tri methylation	26.569	0.0024125	1
GO:0051567	Histone H3-K9 methylation	15.382	0.0072464	1
GO:0042632	Cholesterol homeostasis	13.917	0.0088228	1
GO:0008203	Cholesterol metabolic process	6.9586	0.0088508	1
GO:1902652	Secondary alcohol metabolic process	6.7445	0.0096445	1
GO:0055092	Sterol homeostasis	13.285	0.0096635	1
GO:0034383	Low-density lipoprotein particle clearance	12.707	0.010538	1
GO:0016125	Sterol metabolic process	6.3535	0.0011354	1
