# Supplementary material for: Screening for variable drug responses using human iPSC cohorts
Source: PLoS One. 2025 May 30;20(5):e0323953. doi: 10.1371/journal.pone.0323953 (PMC12124524; doi:10.1371/journal.pone.0323953)
Supplement: S1 Fig — Representative micrographs from six hiPSC lines, plated in 384-well format and stained with pluripotency markers (Oct4, Sox2, Nanog and TRA-181). Scale bar; 100µm. (B) UMAP Embedding of Cell Painting features measured for eight human iPSC lines from phenotypic screens performed on two different dates (2021−02 and 2021−10 on two different imaging systems. hiPSC lines were treated with drugs as described (see Materials and Methods). Cell lines are represented on the graph with different shapes and drugs with different colours. Arrows point to representative drug clusters. Note the high degree of reproducibility of the assay results generated on different dates. (C) PCA analysis of Cell Painting features measured for hiPSC lines in the 2021−02 assay run. Drugs are represented with different colours. (D) PCA analysis of Cell Painting features measured for hiPSC lines in the 2021−10 run. Drugs are represented with different colours. (E) PCA analysis of Cell Painting features measured for hiPSC lines in the 2023−12 run. (Run “2023-12” −18 hiPSC lines). Drugs are represented with different colours. (PDF) [file pone.0323953.s001.pdf]

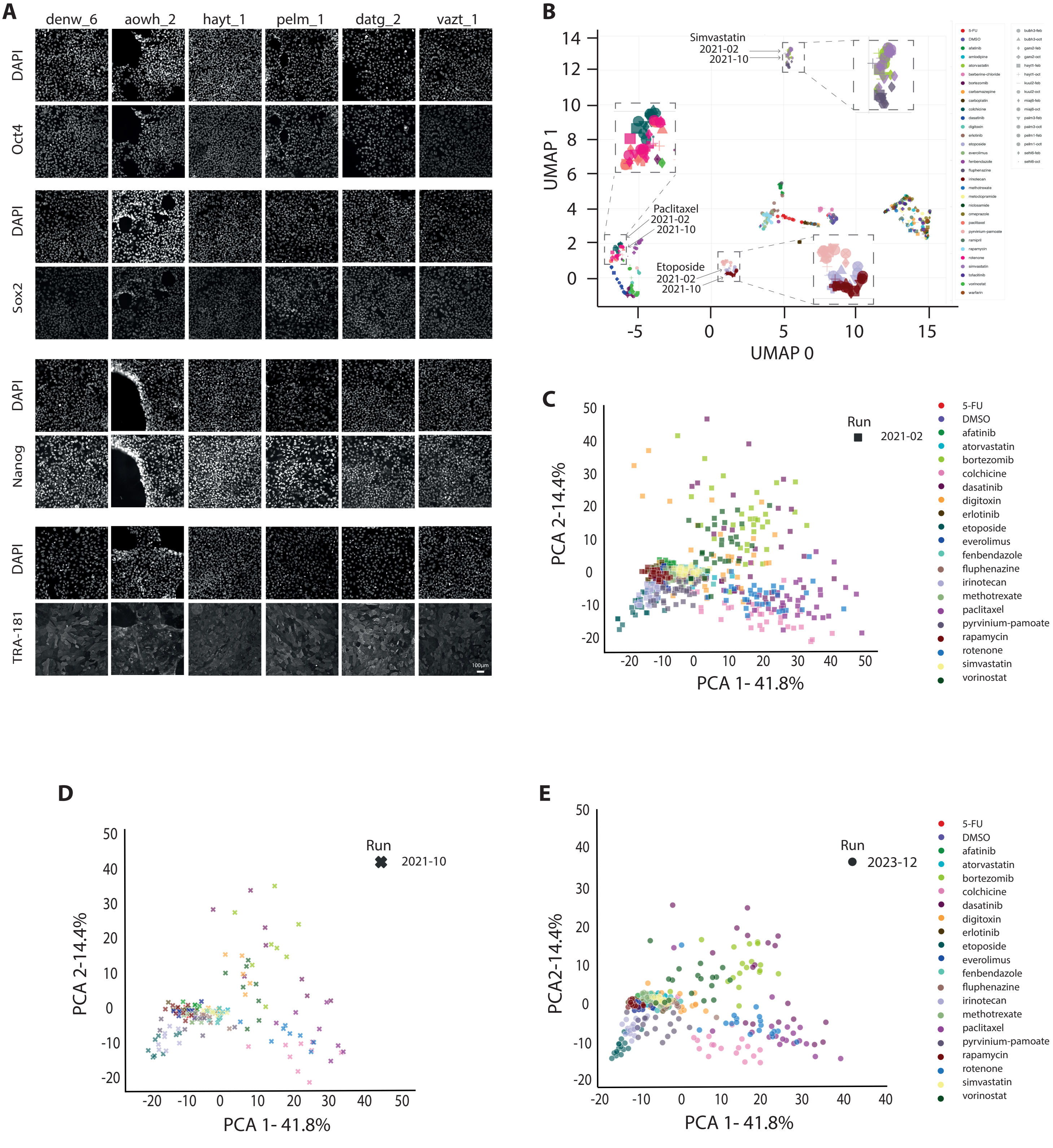

A
denw_6
aowh_2
hayt_1
pelm_1
datg_2
vazt_1
DAPI
Oct4
DAPI
Sox2
DAPI
Nanog
DAPI
TRA-181
100µm
B
Simvastatin
2021-02
2021-10
Paclitaxel
2021-02
2021-10
Etoposide
2021-02
2021-10
UMAP 1
UMAP 0
14
12
10
8
6
4
2
0
-5
0
5
10
15
C
Run
2021-02
PCA 2-14.4%
PCA 1- 41.8%
50
40
30
20
10
0
-10
-20
-20
-10
0
10
20
30
40
50
5-FU
DMSO
afatinib
atorvastatin
bortezomib
colchicine
dasatinib
digitoxin
erlotinib
etoposide
everolimus
fenbendazole
fluphenazine
irinotecan
methotrexate
paclitaxel
pyrvinium-pamoate
rapamycin
rotenone
simvastatin
vorinostat
D
Run
2021-10
PCA 2-14.4%
PCA 1- 41.8%
50
40
30
20
10
0
-10
-20
-20
-10
0
10
20
30
40
50
E
Run
2023-12
PCA2-14.4%
PCA 1- 41.8%
50
40
30
20
10
0
-10
-20
-20
-10
0
10
20
30
40
40
5-FU
DMSO
afatinib
atorvastatin
bortezomib
colchicine
dasatinib
digitoxin
erlotinib
etoposide
everolimus
fenbendazole
fluphenazine
irinotecan
methotrexate
paclitaxel
pyrvinium-pamoate
rapamycin
rotenone
simvastatin
vorinostat
